# Comparison of low-dose maximal-intent versus controlled-tempo resistance training on quality-of-life, functional capacity, and strength in untrained healthy adults: a comparative effectiveness study

**DOI:** 10.1186/s13102-024-00847-z

**Published:** 2024-03-23

**Authors:** Liam T. Pearson, Kai T. Fox, Ashleigh Keenan, David G. Behm, Sam Stuart, Stuart Goodall, Gill Barry

**Affiliations:** 1 Faculty of Health and Life Sciences, Department of Sport, Exercise and Rehabilitation, Northumbria University, Newcastle Upon Tyne, UK; 2 School of Human Kinetics and Recreation, Memorial University of Newfoundland, St. John’s, CA USA

**Keywords:** Activities of daily living, Balance, Independence, Mobility, Untrained

## Abstract

Lack of physical activity is a global issue for adults that can lead to sedentary behaviour and a higher prevalence of health complications and chronic diseases, resulting in reduced quality-of-life (QoL) and functional capacity (FC). A potential strategy to mitigate this inactivity is low-dose resistance training (RT); however, physiological, and psychological responses are limited in evidence. Twenty untrained participants aged 30–60 years old (mean ± SD age 42 ± 7 years, mass 77 ± 13 kg, stature 166 ± 8 cm; 18 females and two males) were recruited and randomly assigned to maximal velocity-intent (MI, *n* = 10) or controlled-tempo (CT, n = 10) RT according to CONsolidated Standards of Reporting Trials (CONSORT) guidelines. Participants attended one training session per week for 6 weeks, consisting of five sets of five repetitions at 60% of one-repetition maximum (1RM) leg press. The interventions differed only during the concentric phase, with MI group pushing with maximal intent, and CT group pushing in a time-controlled manner (3 s). Outcome measures assessed pre- and post-RT included body mass, body mass index (BMI), strength-to-mass ratio, bipedal balance, 6-minute walk test (6MWT), 30-second sit-to-stand (30s-STS), timed up and go (TUG), and leg press 1RM. Time effects were observed for *all* demographics and FC-related outcomes, such as identical reductions in mass and BMI (− 2%), improvements in strength-to-mass ratio (25%) leg press 1RM (22%), 6MWT (3%), and 30s-STS (14%), as well as a 9% improvement in both TUG-clockwise and anticlockwise. Results show low-dose once-weekly RT is effective in improving QoL, FC, and strength in untrained healthy adults, regardless of modality. Positive responses from participants suggest an increased likelihood of consistent participation for low-dose once-weekly RT over more intense modalities. Retrospective ClinicalTrials.gov ID (TRN): NCT06107855, 24/10/2023.

**Supplementary Information:**

The online version contains supplementary material available at 10.1186/s13102-024-00847-z.

## Background

Globally, adults are gradually failing to meet recommended daily physical activity (PA) [1–5] and as a result, are experiencing a rise in body-mass index (BMI) [6–9] and increased prevalence of health complications and chronic diseases [3, 10–13]. These trends are not only accelerating but are linked to age; heightening demand on healthcare services [9, 13–17]. A global surveillance review has recently cited the PA levels and trends are “extremely concerning” [1]. The World Health Organisation (WHO) defines quality-of-life (QoL) as “an individual’s perception of their position in life in the context of the culture and value systems in which they live and in relation to their goals, expectations, standards, and concerns”. Functional Capacity (FC; an individual’s capability to perform tasks and activities necessary for their lives) and PA contribute to all five domains the WHO use to assess QoL: energy and fatigue [18], bodily image and appearance [19], mobility and activities of daily living [20], personal relationships [21, 22], and participation in recreation/leisure activities, highlighting the significance of increasing PA amongst adults.

Regular PA has shown significant associations with an individual’s QoL, with greater levels of PA correlating to better physiological and psychological health [23–26]. Unfortunately, many adults fail to meet basic levels of PA due to various reasons such as perceived time constraints, lack of social support [27], cost [28, 29], and psychological barriers such as nervousness or stigma [30]. One potential solution to address a significant barrier; time, is to explore the physiological and psychological responses of low-dose resistance training (RT), whilst also challenging the mindset and guidelines of how much PA an adult requires to elicit benefits to their QoL [31]. Pain or discomfort has also been identified as common barrier to PA [32], but low-dose RT may help overcome this barrier as lower volumes of RT amount to lower levels of muscular fatigue when compared to higher volumes, even when matched for volume load [33]. Whilst previous research has demonstrated the benefits of RT on strength and neuromuscular function [34], the improvements were observed after high training intensity and volume. Ageing is also associated with a predictable decline in neuromuscular activity [35–38]. RT has been demonstrated to induce relative increases in EMG activity [35–37, 39–41]. Given the links between regular PA and RT for physical and psychological QoL and health benefits, it would be advantageous to recommend PA and RT as a proactive strategy more so than a reactive measure to declining FC or QoL. Early intervention, even during early-middle-age, may mitigate the decline in FC, QoL and cognitive health, with any improvement likely carried over to later life [42, 43].

Maximal-intent (MI), low-dose RT offers benefits that combat barriers to RT, such as time constraints, scalability, and adherence to RT over time; with additional benefits to cardiometabolic conditions via a lower risk of injury compared to high-intensity RT [31, 44–46]. Whilst research on untrained healthy adults is limited, clinical evidence suggests individuals with higher FC are likely to have better QoL and engage in more PA [47, 48]. Moreover, limited evidence exists on the comparisons of maximal- versus controlled-tempo RT to an individual’s FC or QoL [49, 50]. Current research on RT modalities mainly draws on studies of middle-aged and older adults with varying levels of health conditions and functional independence, leaving gaps in the literature regarding the most effective exercise modality for untrained healthy adults [51]. Paterson and Warburton [52] conducted a large-scale systematic review [83,740 participants] for PA in older adults with only two investigations comparing differing RT modalities regarding intent or speed of movement [53, 54], the remaining utilising traditional %1RM-based RT. Similarly, the European Guidelines for Obesity Management in Adults does not stipulate, recommend, or suggest a type or intent of RT, and therefore comparisons of modalities of intent are warranted to help better advise guidelines [55]. Borde, Hortobagyi [56] also reviewed eligible literature and included studies investigating time-under-tension (TUT), a controlled-tempo (CT) RT modality. However, it should be noted that none of the included studies made comparisons between TUT versus other intent- or tempo-related RT modalities, therefore, limited research still exists on comparisons between different RT modalities such as MI or CT.

PA provides both physical and psychological benefits. Whilst an adult may consider themselves healthy now, engaging in PA is critical to maintaining FC in mid-life-adults [57], slowing the decline in QoL [58], may delay cognitive deficits in later years [59], and increases self-efficacy (linked to improved positive physical and mental health status, and satisfaction with life) [60, 61]. Early participation of PA, even during school years, is an important predictor to PA in adulthood. This highlights the importance of adoption of PA as early as possible, regardless of age [62]. According to research by Sequeira, Cruz [63], the top barrier to participation in PA among adults is “lack of time” (55%) and “failure to consider themselves an athlete” scoring 15%. Low-dose RT is a promising solution to these barriers as it requires a short time-commitment whilst also being perceived as a lower barrier-of-entry regarding perceived physical ability when compared to commonly advertised “high-intensity” RT.

The hypothesis of this study was that low-dose MI resistance training would likely lead to greater improvements in QoL, FC, and strength in untrained healthy adults compared to CT due to higher neuromuscular activation elicited by MI exercise.

Therefore, the aims of this study were to:

1. To compare the effects of low-dose Maximal Intent and Controlled Tempo Resistance Training on untrained healthy adults’ quality-of-life, functional capacity, and strength.
2. Qualitatively explore perceptions of Maximal Intent and Controlled Tempo Resistance Training.

## Methods

### Study design

This comparative effectiveness study employed an experimental design using randomised group allocations using an online random number generator. There were no predetermined number of participants for each age category, therefore allocation into age groups was only determined by when the volunteers signed up to the study.

To account for lack of familiarity with equipment, exercises, or tests, the initial session was dedicated to familiarisation. During this session, participants were introduced to the equipment, exercises, and tests they would be required to participate in during the study. Except for the 1RM, which were performed to voluntary volition on both occasions, all other exercise was performed at a moderate intensity.

Strength-to-mass ratio provides a valuable and nuanced perspective into the adaptations that extends beyond absolute strength improvements. Given its relevance to the individual’s body weight, it serves as an indicator of how low-dose RT may influence everyday physical tasks and activities of daily living (ADLs) in untrained individuals, highlighting the potential impact of participating in such an exercise program.

### Study population

Ethical approval was granted from Northumbria University (Approval ID: 3062). Participants aged 30–60 years old (mean age, 42 ± 7 years) were recruited from the local area using social media, word of mouth, emails, and community groups. Prior to their first session, all participants received participant information sheets outlining the study, informed consent forms, and health-screening forms. Participants were advised to not begin any additional RT once the intervention had begun. Table 1 outlines the characteristics of the participants prior to the intervention.

**Table 1:** Pre-intervention participant characteristics

	MI (*n* = 10) (M = 2) (F = 8)	CT (*n* = 10) (M = 0) (F = 10)	*P*
Age (yrs)	41 ± 6	42 ± 7	0.70
Stature (cm)	168 ± 9	163 ± 4	0.12
Mass (kg)	77.1 ± 13.3	77.6 ± 13.3	0.94
BMI	27.3 ± 4.9	26.8 ± 4.9	0.39

Values are mean ± SD. *MI* Maximal-intent, *CT* Controlled-tempo, *M* Male, *F* Female, *yrs* years, *cm* centimetres, *kg* kilograms, *BMI* Body Mass Index

#### Inclusion criteria

Participants were eligible if they were between 30-60 years old, uninjured, had no cardiovascular or neuromuscular conditions, and had not participated in lower-limb RT in the previous 6 months.

#### Exclusion criteria

Participants were excluded if they had either taken part in any lower-limb RT in the last 6 months, had underlying health condition(s) that prevented them from participating in RT, or regularly met or exceeded the UK recommended PA guideline of 150-minutes of moderate to intense PA per week.

#### Randomisation procedure

In the order of their registration, participants were assigned into their respective age categories (30–39 [*n* = 10], 40–49 [*n* = 7], 50–59 [*n* = 3]). Participants were then randomly assigned to either MI or CT training groups. Randomisation was conducted using an online random number generator. Due to limited participants (20), it was not feasible to analyse age-specific training responses. Participants were blinded to the alternative RT modality.

#### Interventions

This study compared MI and CT RT modalities using a unilateral leg press (Perform Better Ltd., Warwickshire, UK). All participants began testing and training sessions with a 10-minute cycling warmup (Wattbike Ltd., Nottingham, UK). The rate of perceived exertion (RPE) scale was employed to monitor and standardise warmup intensity; Participants were granted autonomy to self-select resistance and cadence, with the objective of reaching an RPE of at least 7 at the conclusion of the 10-minute warm up [64, 65]. The study consisted of nine sessions, including two pre-intervention testing sessions in accordance with research by Levinger, Goodman [66], who found in untrained participants, two testing sessions were required to determine a sufficient one-repetition maximum (1RM). The best results from either testing sessions were used for each outcome. Following pre-intervention testing, participants attended one weekly training session for 6 weeks, followed by one post-intervention testing session. Where possible, participants were encouraged to attend their sessions at the same time of the week.

Whilst evidence exists to support single-set exercise improving strength, multi-set exercise still offers significantly superior benefits over single-set in both trained and untrained populations [67]. Therefore, during the training sessions, participants performed two warm up sets of five repetitions on the leg press at 40 and 50% 1RM, then five sets of five repetitions at 60% 1RM. The selected intensity of 60% was chosen as that has been shown to reflect the relative effort at the knee joint needed for both young and older adults when performing actives of daily living such as ascending and descending stairs, and rising from a chair [68]. Both groups were instructed to control the eccentric phase of the leg press over three-seconds. The interventions differed only during the concentric phase, with both groups following a metronome app [69], that controlled the eccentric phase to three-seconds. The MI group was instructed and encouraged to deliberately commit to exerting maximal effort and force during the concentric phase of the leg press with the intention of achieving the greatest velocity possible, while the CT group followed the metronome for both eccentric and concentric phases. To assist with standardisation and minimise variability in movement patterns, both groups were supported by a metronome (during both concentric and eccentric phases of the CT group, and only during the eccentric phase of the MI group), as well as a large colour-display that indicated concentric and eccentric phases, verbal guidance from a smartphone app, and verbal encouragement specific to their training group from the supervising researcher.

### Outcome measures

#### Body mass

Body mass was measured using a calibrated weighing scale (Seca 704 s, GmBH & Co Kg, Hamburg, Germany) to ensure consistency, participants were weighed at the same time of day.

#### Strength-to-mass ratio

Strength-to-mass was calculated by dividing the individuals leg press 1RM by their body mass.

#### Balance

Balance was assessed using a calibrated balance system (Biodex Balance System™, Biodex Medical Inc., New York, USA). After one trial set, three 20-second tests were performed using the Testing > Postural Stability pre-set. Based on pilot testing, a platform setting of six was selected as it was deemed repeatable enough without exposing participants to the greater risk of injury that may occur at more difficult platform settings. During the tests, balance overall (BalanceO) scores, as well as anterior-posterior (BalanceAP), and medial-lateral (BalanceML) scores, were recorded. All testing was performed on two legs (bipedal balance).

#### 6-minute walk test (6MWT)

Participants were asked to walk and turn on a 30-m distance at a brisk walking pace on an indoor running track for 6 minutes. Participants were blinded to time-remaining and without any verbal encouragement, to better represent their natural gait speed. Distance travelled was recorded in metres.

#### 30-second sit-to-stand (STS)

Participants were asked to sit on a chair with a solid back and arm rests, as recommended by the Centers for Disease Control and Prevention (CDC) [70], with their arms crossed over their chest and feet flat on the floor throughout the test (to avoid ‘rocking’). Participants were asked to perform as many repetitions from a seated to standing position as possible in 30 seconds.

#### Timed up and go (TUG)

Participants were encouraged to rise from the same chair as the STS, with arms crossed over their chest, and to walk as quickly as possible around a marked position three metres in front of the chair. Both clockwise (TUGc) and anticlockwise (TUGa) directions were attempted with a one-minute rest in between, and the direction attempted first was randomised at each testing session. Time was recorded from the moment the participants weight left the chair to when it was placed back on.

#### Leg press one-repetition maximum (1RM)

Participants were taken through a progressive warmup which allowed for discussion and practise of minimum knee flexion (90°) to be considered a standardised repetition [71, 72]. Participants were encouraged to hold onto the handles to anchor themselves into the chair, to brace, and were asked to maintain contact with their head, shoulders, back, and pelvis against the chair during the repetitions. Weight lifted was progressed in lower increments as the participant began reaching their 1RM.

Recovery between attempts was standardised to two-minutes, with the aim of a 1RM to be achieved within eight or fewer sets in coordination with research that reviewed trained individuals may get a 1RM within three to five repetitions [73], therefore flexibility was given as participants were untrained. Testing ended when participants either reached their 1RM or failed two consecutive attempts, in which case the previous successful lift was recorded. All testing and training were conducted unilaterally.

### Statistical analysis

Data are presented as mean ± SD. Statistical analysis was performed using the SPSS Statistics (v26.0, IBM Corp., Chicago, IL). G*Power [74] was used to determine sample size using effect size of 0.5, alpha at 0.05, and power at 0.8, for a two-group design with two measurements “ANOVA: Repeated measures, within-between interaction.” Assuming moderate correlation among measures (0.5) and sphericity (nonsphericity correction of 1), G*Power calculated a need for 12 participants per group. Testing for normality was conducted on all dependent variables and Shapiro-Wilk’s output was used due to the small sample size [75], and where any dependent variable departed significantly from normality, visual examination of the histogram and QQ plot took place before any further parametric tests were conducted. The five assumptions of a two-way ANOVA were also checked before further analysis was conducted [76]; Mauchly’s test of sphericity was not reported as no within-subject factor had more than two (pre- and post-intervention) categories (timepoints). A two-sample t-test was then conducted to analyse differences in baseline values, and a 2 × 2 two-way repeated-measures ANOVA was used to analyse the effect of the two training modalities (MI vs. CT) over two timepoints (pre- and post-intervention), with *p* ≤ 0.05 deemed to be statistically significant. As previously mentioned with 20 participants of whom only two were men, age and sex differences could not be partitioned. Effect sizes were calculated using Cohen’s D (where > 0.2: trivial, 0.2 - < 0.5: small, 0.5 - < 0.8: moderate, > 0.8: large magnitude difference [77]), and observed power is denoted as P (obs). Partial Eta-squared (ηp2) values are reported for main effects and overall interactions representing small (0.01 ≤ ηp2 < 0.06), medium (0.06 ≤ ηp2 < 0.14) and large (ηp2 ≥ 0.14) magnitudes of change. If an interaction effect was found, due to small sample size, a Bonferroni post-hoc test was chosen to find where the differences were present. To assess 1RM reliability across the two pre-intervention tests, both the Intraclass Correlation Coefficient (ICC) and Coefficient of Variation (CV%) were calculated. The ICC was determined using a Two-Way Random model with Absolute Agreement, using SPSS Statistics (v26.0, IBM Corp., Chicago, IL). The CV% was calculated using the formula (Standard Deviation / Mean) * 100 on Microsoft Excel (v2312, Microsoft Corporation, Redmond, WA).

### Qualitative analysis

To better understand participants’ perceptions of the training and its impact on their QoL, FC, and strength, a focus group was conducted. All participants were invited to attend a focus group sessions held at the Sport Science facilities at Northumbria University, with those unable to attend in-person offered to join online. The aim was to gather detailed feedback of the participants’ experience with their respective intervention. Those unable to attend in-person or online were provided the same set of open-ended questions, curated by the research lead and primary investigator (Appendix 1), with the scope of fostering in-depth discussions about the individual protocols perceived effectiveness, applicability, and impact on participants’ overall health and wellbeing. The entirety of the focus group, both in-person and online was recorded (with participants’ consent). Subsequently, the recordings were transcribed verbatim by the research lead, utilising Google Text-to-Speech (v0.2.7, Alphabet Inc., California, U.S.), supplemented by manual transcription.

Thematic analysis was then carried out to review the transcripts to identify codes highlighting recurring words, phrases, and sentiments; broader themes were then derived from these codes to gather the overall experiences and outcomes reported by the participants. After independent analysis, findings were discussed with the primary investigator. Any discrepancies or disagreements between reviewers were resolved through consultation of a third reviewer. Parentheses indicate participant ID and RT modality.

## Results

Participant characteristics were well-matched between groups at baseline (Table 1). Two-way ANOVA analysis can be found in the Supplemental Data File (Appendix 2), along with the Shapiro-Wilk’s output (Appendix 3). The ICC for the average measurements over both 1RM pre-testing sessions was .963 (95%CI .239 to .992, *F(19,19)* = 89.68, *p* < .001), indicating excellent reliability between tests. The CV% for the first and second pre-intervention sessions were 26.83 and 26.16%, respectively, demonstrating consistency across measurements.

**Fig. 1:**
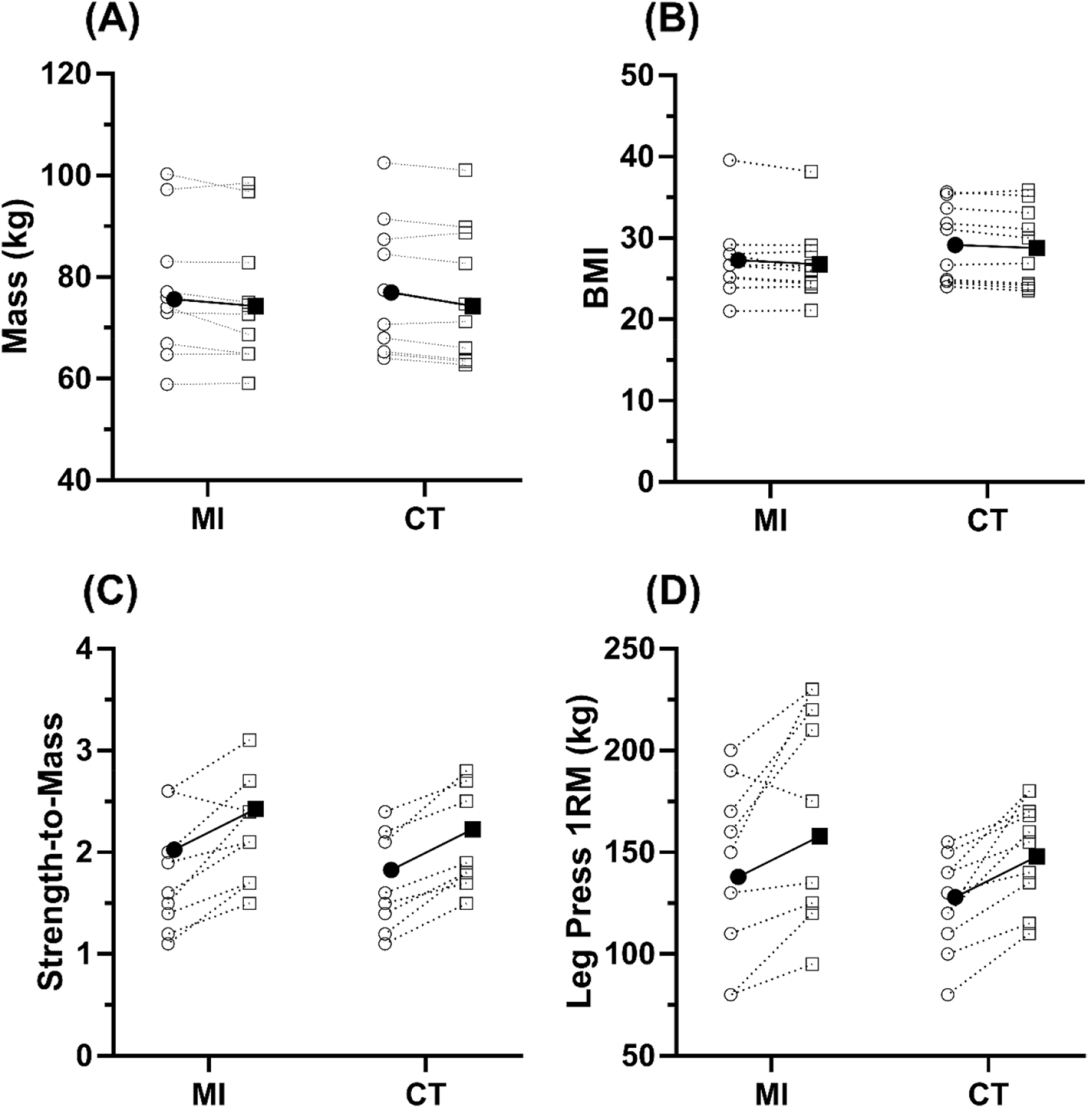
Demographic characteristics pre and post training. MI = Max-intent, CT = controlled-tempo; Panel **A** shows body mass, panel **B** shows body mass index (BMI), panel **C** shows strength to mass ratio, and panel **D** shows leg press 1 repetition maximum. Unfilled symbols show individual pre to post changes, filled symbols show the mean response

**Fig. 2:**
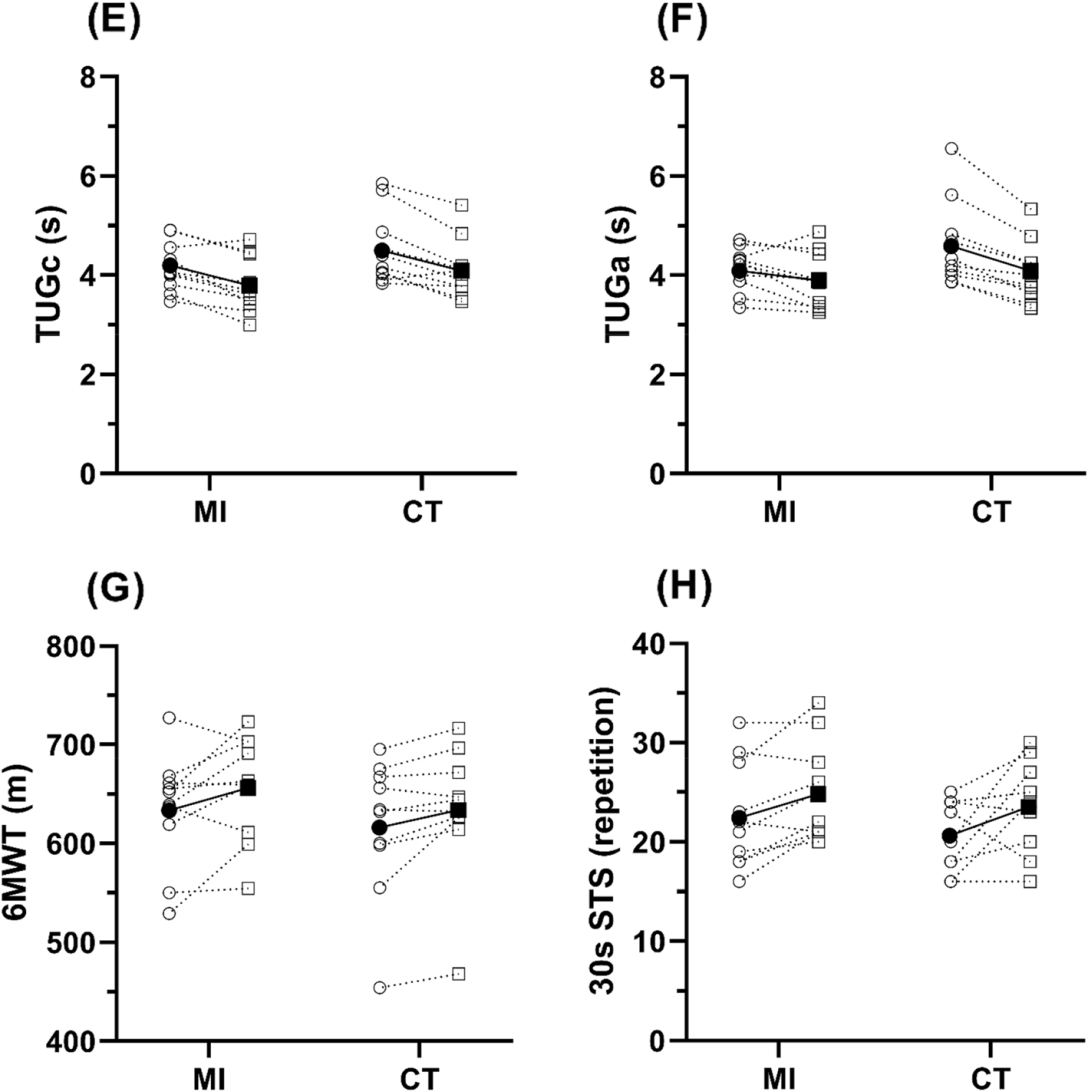
Functional Capacity pre and post training. Panel **A** shows the timed up and go clockwise (TUGc), panel **B** shows the TUG anticlockwise (TUGa), panel **C** shows the six-minute walk test (6MWT), and panel **D** shows the 30 second sit to stand (30 s STS). Unfilled symbols show individual pre to post changes, filled symbols show the mean response. * represents statistical significance

**Fig. 3:**
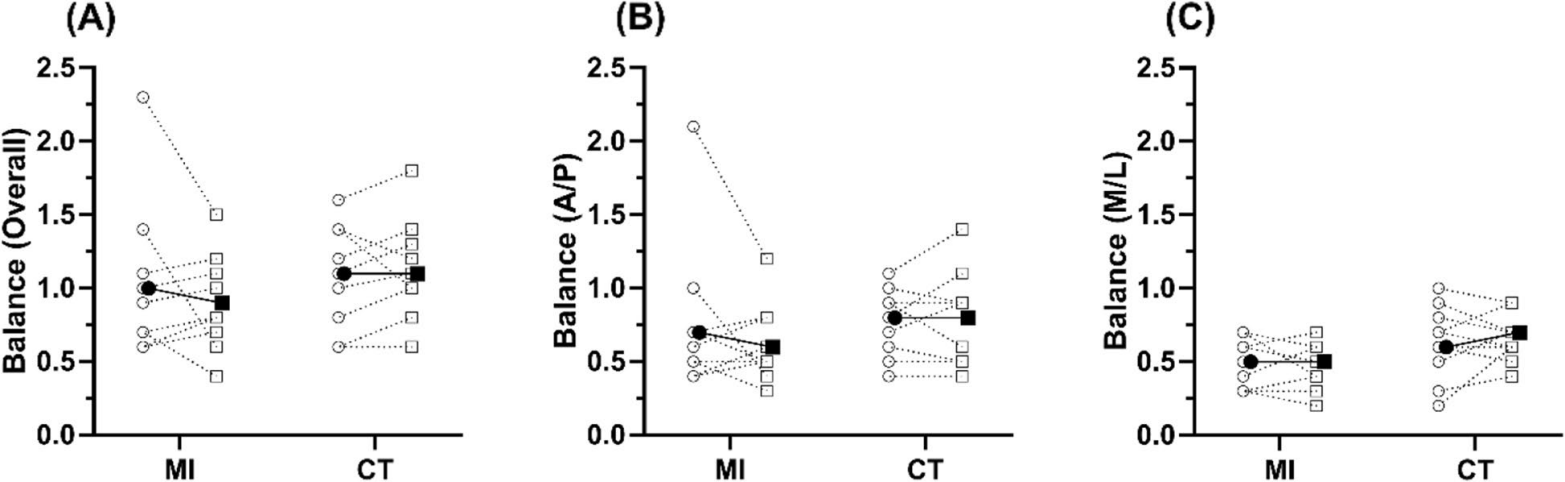
Balance pre and post training. Panel **A** shows overall balance performance, panel **B** shows anterior-posterior sway (A/P), and panel **C** shows medial-lateral sway (M/L). Unfilled symbols show individual pre to post changes, filled symbols show the mean response

MI and CT produced similar changes in mass, and therefore BMI (MI Δ: − 1.7 ± 2.5% vs. CT Δ: − 1.6 ± 1.5%, Fig.1 A & B, respectively), with strength and strength-to-mass ratio improving by near a quarter in both groups (Strength: MI Δ: 22.1 ± 19.2% vs. CT Δ: 22.7 ± 14.2%, Fig. 1D; Strength-to-Mass: MI Δ: 24.6 ± 21.7 vs. CT Δ: 24.6 ± 13.6%, Fig. 1C). TUG times showed large, yet similar, improvements following both training groups when turning clockwise (MI Δ: − 8.9 ± 6.5% vs. CT Δ: − 8.9 ± 6.4%, Fig. 2E), however, there was a greater improvement in CT when turning anticlockwise (MI Δ: − 5.8 ± 8.5% vs. CT Δ: − 11.5 ± 4.6%, Fig. 2F). Likewise, both groups showed similar improvements in 6MWT, and 30-second STS performance (Fig. 2G & H, respectively) and balance scores showed very little change between groups, with high levels of variability (Fig. 3).

### Recruitment and attrition

All 20 participants (MI: *n* = 10, CT: *n* = 10) completed the intervention, without injury or adverse events.

### Demographic characteristics

A time effect was evident for all demographic outcomes: A reduction in body mass (*F(1,9)* = 21.32, *p* < 0.01, *η*_*p*_^*2*^ *= .703*), and BMI was evident (*F(1,9)* = 10.41, *p* = 0.01, *η*_*p*_^*2*^ *= .536*), as well as an increase in the strength-to-mass ratio (*F(1,9)* = 36.12, *p* < 0.01, *η*_*p*_^*2*^ *= .801*), and 1RM (*F*(1,9) = 21.32, *p* < 0.01, *η*_*p*_^*2*^ = .703) post-intervention. However, no group or interaction effects were evident between groups for any demographic outcome (*P* ≥ 0.25; all effect sizes ≤0.11).

### Functional capacity

Across all FC-related outcomes, only a time effect was observed: A reduction in TUGc (*F(1,9)* = 30.27, *p* < 0.01, *η*_*p*_^*2*^ = .771), and TUGa times (*F(1,9)* = 19.17, *p* < 0.01, *η*_*p*_^*2*^ = .681), as well as an increase in 6MWT (*F(1,9)* = 12.35, *p* < 0.01, *η*_*p*_^*2*^ = .578), and 30sSTS (*F(1,9)* = 7.71, *p* = 0.02, *η*_*p*_^*2*^ = .461) were observed post-intervention; with an interaction effect present for TUGa (MI Δ: − 0.2 ± 0.4 vs. CT Δ: − 0.6 ± 0.3 s; *F(1,9)* = 7.44, *p* = 0.02, *η*_*p*_^*2*^ = .453). However, post-hoc tests did not reveal any differences across multiple comparisons. After observing the lack of differences between groups at baseline or post-exercise, combined mean differences post-exercise were determined (TUGa: 0.4 ± 0.1, 6MWT: 5.1 ± 13.2, 30sSTS: 0.5 ± 2.3).

### Balance

A group effect for BalanceML was the only observation noted across all balance parameters, with CT performing marginally better than MI (MI Δ: 0.0 ± 0.1 (− 0.7 ± 28.5%) vs. CT Δ: 0.0 ± 0.2 (22.6 ± 66.4%); *F(1,9)* = 5.10, *p* = 0.05, *η*_*p*_^*2*^ = .362). No time or interaction effects were found across any balance-related outcomes.

### Focus group

#### Exercise intensity

When asked about their perceptions of exercising at 60% intensity, similar trends were observed, with comments for example:

- *“About right”* – (P3, MI)
- *“Was feeling it by the last set, so it felt appropriate”* – (P7, CT)
- *“Definitely felt sore after the first two sessions, but got better after that, so I’d say 60% was about right”* – (P1, CT)
- *“I could have done more, but maybe would have been too sore the next day, so I was happy with 60%”* – (P14, CT)
- *“Not too taxing”* – (P4, MI)

#### Controlled movement speed

Regarding their opinions on the three-second control, there were mixed responses, as exemplified by the following comments:

- *“I just wanted to push hard”* – (P11, CT)
- *“Was difficult to time at first”* – (P17, CT)
- *“Unsure how or even if I’d do the slow movement in a regular gym without Liam there”* – (P18, CT)
- *“Eventually began to find it easier”* – (P14, CT)

#### Increasing intensity

Participants were also asked if there was a point during the intervention where they could have increased resistance, and the majority agreed *“the three-to-four-week mark”*, stating they felt the sessions were getting easier, so must have been getting stronger, and therefore would have liked an increase*. However,* one participant in the CT group felt they could *“at no point could have increased the weight”* – (P18, CT).

#### Quality-of-life changes

When participants were asked about any comments regarding changes in QoL, a notable response from one participant said she now finds it easier to *“pick my child up completely unassisted”* (P14, CT) and could not emphasis enough how this has improved her QoL and ADLs. Other QoL-related comments were:

- “*Yes, I felt stronger which made me feel better”* – (P3, MI)
- *“My energy levels definitely felt whilst participating in this study”* – (P8, MI)
- *“Felt a good sense of achievement when my 1RM went up”* – (P18, CT)
- *“I’ve started to feel my flexibility is actually better since doing this”* – (P15, CT)
- *“Definitely felt more positive”* – (P19, CT)
- *“Hard to tell, but all my tests improved so I feel better for that”* – (P5, MI)
- *“Definitely felt like my legs have more ability to push harder now when I visit some trial walks etc.”* and *“I noticed my thighs looked thinner and that’s improved my self-confidence”* – (P19, CT)

When asked whether participants felt they could notice improvements in their FC in ADLs, most participants agreed they could not, regardless of their improvements in pre-post change scores. It is possible that this may be attributed to participants already scoring well for balance and 6MWT, and older or clinical populations may perceive a more noticeable difference.

#### Perceived behavioural changes to continue low-dose RT

When asked whether they would continue a low-dose intervention, participants unanimously agreed they would, with comments such as:

- *“Yes, because despite not ‘feeling’ the walking benefits, I definitely feel better in general”* – (P12, CT)
- *“I’ve actually joined a gym now because of this”* – (P19, CT)
- *“I’m definitely going to keep this up as it’s something I’m fortunate I can do during my lunch break”* – (P7, MI)
- *“I liked this minimalist test, and I also definitely want to continue as it doesn’t take long”* – (P12, CT)
- *“This has given me the motivation, now I know I don’t have to do too much, as I’m not really a ‘gym person’”* – (P8, MI)
- *“I hate having to spend ‘hours in the gym’ so I’m encouraged I can still get benefits from shorter sessions”* – (P18, CT)
- *“Even though I think I’ve a dodgy knee, I actually think this low dose helps my knee as it definitely feels better without it hurting like it usually does when I try going to the gym or signing up to personal training sessions”* – (P5, MI)

We saw a positive trend occurring throughout the focus group in relation to low-dose RT irrespective of training group. The element of time is a key factor in that people enjoyed the aspect of only having to do one session per week and felt this could easily fit into their lifestyle as opposed to more intense training.

When asked if there was anything they would have changed, some participants expressed concern about the positioning of the weights being above them when using the leg press. Specifically, some found it intimidating, noting it as something to consider if working with older adults or clinical populations. Other concluding comments were:

- “*All good from me, I actually want to learn more about this low dose”* – (P15, CT)
- *“Now I’ve been told there is another type of group, I’d like to try that to see which one suits me better”* – (P19, CT)
- *“I would be happy to participant in such a study again but would like to change the position of the timing-app as I struggled to see it when it was offset to the side of me, and unsure how I’d use a timing-app in a regular gym”* – (P12, CT)

In summary, participants reported feeling generally psychological better and experiencing improvements in strength, FC and QoL from low-dose RT, regardless of training group. This suggests indication of both qualitative and quantitative improvements, although larger sample sizes are needed to strengthen statistical power. The overall positive responses suggest an increased likelihood of an intention to consistently participate that a low-dose RT presents over more intense traditional-RT.

## Discussion

### Overview

The current UK physical activity guidelines [78], whilst encouraging vigorous activity, lack specifics in regards to sets, repetitions, %1RM, or number of exercises. Low-dose resistance training improved across *all* demographic and FC related outcomes; the significance of strength-to-mass has well-established impact across the lifespan of humans across all levels of ability, such as in ADL’s of ageing men [79], locomotion efficiency [80], bone mineral density [81], and as a combatant to sarcopenia [82]. Both groups observed a noticeable improvement in 6MWT after only six sessions (MI Δ (%): 23 ± 36%, CT Δ (%): 18 ± 22%, Fig. 2C), and given the significant association between walking speed and early predictor of mortality [83, 84], low-dose RT seems to be showing evidence to combat this and therefore holds merit to investigate further in those who’s walking speed are trending towards the 0.82 m/s threshold.

The purpose of this study was twofold: to initiate comparative research between low-dose maximal-intent (MI) and controlled-tempo (CT) RT modalities on QoL, FC, and strength in untrained healthy older adults; and to qualitatively explore their perceptions of the differing training modalities in relation to the same outcomes. Whilst improvements were evident across both groups post-training, there was a lack of difference between the training groups. Nevertheless, data suggests both low-dose MI and CT appear viable considerations that combat barriers to exercise, whilst also showing improvements in QoL- and FC-related outcomes. Additionally, qualitative information gathered from the post-intervention focus group revealed participants self-reported a high level of satisfaction regardless of training modality, as well as reporting a willingness to continue low-dose RT.

Collectively, these results demonstrate how engaging in only one session of low-dose RT a week can elicit positive benefits in healthy adults QoL, FC, and strength. However, the role of ‘intent’ did not appear to produce an enhanced improvement. Given the innovative nature and focus on low-dose RT in untrained healthy adults of this research, direct comparisons with existing knowledge are challenging. Therefore, the results of this study offer preliminary insights and should only be inferred when drawing comparisons to existing research in the field of QoL and FC, with consideration for potential differences in age and health status of comparative study populations. For example, research by Sousa and Sampaio [85] found leg press 1RM (kg) to improve by over 50% in older adults (mean age 73 ± 6 years, *n* = 20) compared to ~ 20% observed in the present study (mean age 42 ± 7); A likely cause for this is thatSousa and Sampaio [85] conducted strength-training 3 days per week over 12-weeks, with training intensity being recalibrated after 1RM testing at weeks five and nine, compared to once-weekly over six-weeks with no increased in intensity in the present study. Brandon, Boyette [86] observed a 0.3 second improvements in TUG in older adults (age range 60–86 years) over 24-months, with 3 days per week of 11 exercises at three sets of eight repetitions. Whereas this study found improvements of 0.24 and 0.55 second from baseline in MI and CT, respectively, in only six-weeks of once-weekly RT. In contrast, similar improvements in 6WMT were observed in this study to that of Henwood and Taaffe [87], however, what was conducted was with older adults aged 65–84 years who performed six exercises for three sets of 10 repetitions, for 16 sessions, versus only six sessions in this study. The resistance also increased when Henwood and Taaffe [87]‘s participants could complete ≥eight repetitions on their third set, compared to no increase in load in this intervention. Based on the findings within this study, one exercise (leg press), once weekly, with less repetitions and with lower %1RM, demonstrates efficacy in eliciting similar benefits to that of higher amounts of exercises, loads, and volumes.

With existing research supporting movement speed as a major factor for improving neuromuscular activity such as increase excitability, and inhibition in the corticospinal tract [88], these findings suggest variables such as volume, intensity, and fatigue, also need to be considered. The low-volume, moderate-intensity nature of exercise employed in this study results in minimal exercise-induced fatigue among participants.

The mean participant age, 41.5 years old, likely influenced the findings of this research. It is plausible to suggest their physiological capabilities were not yet significantly deteriorated, thus limiting the detectability of improvement from employing such a low-dose methodology. Their pre-intervention test scores suggest minimal age-related decline in function and QoL and could explain the minimal improvements observed. It is unlikely this age range, although possible, exhibit deteriorations in gait, balance, or strength, nor are they likely suffering significant sarcopenia or reductions in androgenic hormones. However, that is not to say research of this nature is not warranted, as there are adults that fall under these categories.

The continuous supervision observed in this study likely influence participants’ effort, thereby influencing the results. It is important to acknowledge individuals exercising unsupervised may not achieve the same degree of success. Therefore, supervision should be considered when interpreting and applying these findings.

### Study limitations

The limitations of this study are largely attributed to the low sample size. Despite the potential lower statistical power, our data still show the positive impact of low dose RT. For TUG and sit-to-stand assessments, a chair with a fixed height was used. Chair height should ideally be adjustable, allowing it to be proportional in height to the participants lower-limb length to allow for more personalised testing [89]. To gain greater insight of QoL outcomes, it is recommended for future research that, in addition to focus groups, questionnaires such as the SF36-II [90], or SF12 [91] should be included. This should allow for more nuanced perspectives on QoL-related outcomes and to supplement data gathered from focus groups, resulting in richer insights into the experiences of the participants.

It is likely participants’ activity levels and ADL improved during the intervention as a result of starting an exercise study. This was not accounted for in this research. Future studies are recommended to attempt to capture this data by means of daily activity monitors.

## Conclusion

The positive findings within this study indicate low-dose RT is viable, irrespective of modality, to consider for QoL, FC, and strength in untrained healthy adults. Insight such as this could be inferred to clinical or physiotherapy settings when developing rehabilitation programs, due to the low-volume low-intensity nature of programmes prescribed in the early stages of recovery. Further larger scale research is necessary to confirm these preliminary findings and to explore the potential benefits in older populations, in addition to individuals with differing health-conditions; As for example, if older adults were to elicit similar relative changes as observed in this study, the significance of those findings to the older population would likely be greater intrinsically.

### Supplementary Information

**Supplementary Material 1.**

**Supplementary Material 2.**

**Supplementary Material 3.**

## Data Availability

The datasets used and/or analysed during the current study are available from the corresponding author on reasonable request.
