# Supplementary material for: Comparison of low-dose maximal-intent versus controlled-tempo resistance training on quality-of-life, functional capacity, and strength in untrained healthy adults: a comparative effectiveness study
Source: BMC Sports Sci Med Rehabil. 2024 Mar 23;16:72. doi: 10.1186/s13102-024-00847-z (PMC10961002; doi:10.1186/s13102-024-00847-z)
Supplement: Supplementary file 1 — Supplementary Material 1. [file 13102_2024_847_MOESM1_ESM.docx]

## Appendix 1

### Questions Specific to The Training

- How did you find the CT training?
- How did you find the MI training?
- Did you find it hard to control the movement down for 3 seconds and up again for 3 seconds at 60% 1RM in the CT group?
- Did you find it hard to control the movement down for 3 seconds and up again for 3 seconds at 60% 1RM in the MI group?
- Was 60% enough for the MI group?
- Was 60% enough for the CT group?
- Did you feel there was a point in the intervention over the 6-weeks when you found the exercise to become noticeably easier / more adapted to?
- Did you feel once a week was sufficient?
- Did you feel the number of reps performed was sufficient?
- How did you find using/adapt to the leg press with both feet moving independently?

### Questions Relating to Quality-of-Life

- How did you feel your strength changed after the intervention?
- Did you think your walking speed or balance changed?
- Have you noticed any changes around your general lifestyle/ADL’s?
- Would you like to implement this kind of strength training into your routine?
  - Do you think you will?

### General Comments

- Is there anything that you would like to have seen improved with the study?
