# Supplementary material for: Comparison of low-dose maximal-intent versus controlled-tempo resistance training on quality-of-life, functional capacity, and strength in untrained healthy adults: a comparative effectiveness study
Source: BMC Sports Sci Med Rehabil. 2024 Mar 23;16:72. doi: 10.1186/s13102-024-00847-z (PMC10961002; doi:10.1186/s13102-024-00847-z)
Supplement: Supplementary file 2 — Supplementary Material 2. [file 13102_2024_847_MOESM2_ESM.docx]

| **Appendix 2.**  Pre-Post Intervention Results | | | | | | | | | | | |
| --- | --- | --- | --- | --- | --- | --- | --- | --- | --- | --- | --- |
|  |  | **Pre-intervention** | **Post-intervention** | **Δ** | **Δ (%)** | **Effect Size** |  | **F** | **Sig** | **P(obs) Power ^a^** | **Partial Eta Squared** |
| **Demographic** |  |  |  |  |  |  |  |  |  |  |  |
| **Mass, kg** | MI: | 77.1 ± 13.3 | 75.8 ± 13.3 | -1.4 ± 2.0 | -1.7 ± 2.5% | 0.09 |  |  |  |  |  |
|  | CT: | 77.6 ± 13.3 | 76.4 ± 13.4 | -1.2 ± 1.2 | -1.6 ± 1.5% |  |  |  |  |  |  |
|  |  |  |  |  |  |  | Group | 1.49 | 0.25 | 0.20 | 0.14 |
|  |  |  |  |  |  |  | Time | 21.32 | <0.01* | 0.98 | 0.70 |
|  |  |  |  |  |  |  | Group*Time | 0.05 | 0.82 | 0.06 | 0.01 |
| **BMI** | MI: | 27.3 ± 4.9 | 26.8 ± 4.6 | -0.5 ± 0.7 | -1.7 ± 2.5% | 0.11 |  |  |  |  |  |
|  | CT: | 29.2 ± 4.8 | 28.8 ± 4.9 | -0.4 ± 0.5 | -1.6 ± 1.5% |  |  |  |  |  |  |
|  |  |  |  |  |  |  | Group | 1.50 | 0.25 | 0.20 | 0.14 |
|  |  |  |  |  |  |  | Time | 10.41 | 0.01* | 0.82 | 0.54 |
|  |  |  |  |  |  |  | Group*Time | 0.07 | 0.79 | 0.60 | 0.01 |
| **Strength-to-mass** | MI: | 1.8 ± 0.5 | 2.2 ± 0.5 | 0.4 ± 0.3 | 24.6 ± 21.7% | 0.01 |  |  |  |  |  |
|  | CT: | 1.6 ± 0.4 | 2.0 ± 0.5 | 0.4 ± 0.2 | 24.6 ± 13.6% |  |  |  |  |  |  |
|  |  |  |  |  |  |  | Group | 0.62 | 0.45 | 0.11 | 0.06 |
|  |  |  |  |  |  |  | Time | 36.12 | <0.01* | 1.00 | 0.80 |
|  |  |  |  |  |  |  | Group*Time | 0.05 | 0.82 | 0.06 | 0.01 |
| **Leg Press 1RM, kg** | MI: | 138.0 ± 42.9 | 166.5 ± 52.3 | 28.5 ± 27.3 | 22.1 ± 19.2% | 0.08 |  |  |  |  |  |
|  | CT: | 124.5 ± 23.4 | 151.3 ± 25.3 | 26.8 ± 15.8 | 22.7 ± 14.2% |  |  |  |  |  |  |
|  |  |  |  |  |  |  | Group | 1.49 | 0.25 | 0.20 | 0.14 |
|  |  |  |  |  |  |  | Time | 21.32 | <0.01* | 0.98 | 0.70 |
|  |  |  |  |  |  |  | Group*Time | 0.05 | 0.82 | 0.06 | 0.01 |
| **Functional Capacity** |  |  |  |  |  |  |  |  |  |  |  |
| **TUGc, s** | MI: | 4.2 ± 0.5 | 3.8 ± 0.6 | -0.4 ± 0.3 | -8.9 ± 6.5% | 0.19 |  |  |  |  |  |
|  | CT: | 4.5 ± 0.7 | 4.1 ± 0.6 | -0.4 ± 0.3 | -8.9 ± 6.4% |  |  |  |  |  |  |
|  |  |  |  |  |  |  | Group | 1.36 | 0.27 | 0.18 | 0.13 |
|  |  |  |  |  |  |  | Time | 32.27 | <0.01* | 1.00 | 0.77 |
|  |  |  |  |  |  |  | Group*Time | 0.22 | 0.65 | 0.07 | 0.24 |
| **TUGa, s** | MI: | 4.1 ± 0.4 | 3.9 ± 0.6 | -0.2 ± 0.4 | -5.8 ± 8.5% | 0.93 |  |  |  |  |  |
|  | CT: | 4.6 ± 0.9 | 4.1 ± 0.6 | -0.6 ± 0.3 | -11.5 ± 4.6% |  |  |  |  |  |  |
|  |  |  |  |  |  |  | Group | 1.141 | 0.31 | 0.16 | 0.11 |
|  |  |  |  |  |  |  | Time | 19.17 | <0.01* | 0.97 | 0.68 |
|  |  |  |  |  |  |  | Group*Time | 7.44 | 0.02* | 0.68 | 0.45 |
| **6MWT, m** | MI: | 633.7 ± 57.4 | 656.5 ± 53.5 | 22.8 ± 35.9 | 3.8 ± 5.9% | 0.17 |  |  |  |  |  |
|  | CT: | 616.6 ± 70.7 | 634.3 ± 67.1 | 17.7 ± 21.8 | 3.0 ± 3.9% |  |  |  |  |  |  |
|  |  |  |  |  |  |  | Group | 0.37 | 0.56 | 0.08 | 0.04 |
|  |  |  |  |  |  |  | Time | 12.35 | <0.01* | 0.88 | 0.58 |
|  |  |  |  |  |  |  | Group*Time | 0.12 | 0.74 | 0.06 | 0.01 |
| **30sec STS, reps** | MI: | 22.6 ± 5.4 | 25.0 ± 5.0 | 2.4 ± 2.5 | 12.1 ± 12.2% | 0.13 |  |  |  |  |  |
|  | CT: | 20.8 ± 3.6 | 23.7 ± 4.6 | 2.9 ± 4.8 | 16.0 ± 25.5% |  |  |  |  |  |  |
|  |  |  |  |  |  |  | Group | 0.48 | 0.51 | 0.10 | 0.05 |
|  |  |  |  |  |  |  | Time | 7.71 | 0.02* | 0.70 | 0.46 |
|  |  |  |  |  |  |  | Group*Time | 0.11 | 0.75 | 0.06 | 0.01 |
| **Balance** |  |  |  |  |  |  |  |  |  |  |  |
| **BalanceO** | MI: | 1.0 ± 0.5 | 0.9 ± 0.3 | -0.1 ± 0.4 | -2.9 ± 30.3% | 0.44 |  |  |  |  |  |
|  | CT: | 1.1 ± 0.4 | 1.1 ± 0.3 | 0.0 ± 0.2 | 5.9 ± 19.7% |  |  |  |  |  |  |
|  |  |  |  |  |  |  | Group | 1.13 | 0.32 | 0.16 | 0.11 |
|  |  |  |  |  |  |  | Time | 0.41 | 0.54 | 0.09 | 0.04 |
|  |  |  |  |  |  |  | Group*Time | 0.81 | 0.39 | 0.13 | 0.08 |
| **BalanceAP** | MI: | 0.7 ± 0.5 | 0.6 ± 0.3 | -0.1 ± 0.4 | 0.1 ± 40.5% | 0.47 |  |  |  |  |  |
|  | CT: | 0.8 ± 0.2 | 0.8 ± 0.3 | 0.0 ± 0.2 | 3.3 ± 22.0% |  |  |  |  |  |  |
|  |  |  |  |  |  |  | Group | 0.70 | 0.42 | 0.12 | 0.07 |
|  |  |  |  |  |  |  | Time | 0.43 | 0.53 | 0.09 | 0.05 |
|  |  |  |  |  |  |  | Group*Time | 0.97 | 0.35 | 0.14 | 0.10 |
| **BalanceML** | MI: | 0.5 ± 0.2 | 0.5 ± 0.1 | 0.0 ± 0.1 | -0.7 ± 28.5% | 0.30 |  |  |  |  |  |
|  | CT: | 0.6 ± 0.2 | 0.7 ± 0.2 | 0.0 ± 0.2 | 22.6 ± 66.4% |  |  |  |  |  |  |
|  |  |  |  |  |  |  | Group | 5.10 | 0.05* | 0.52 | 0.36 |
|  |  |  |  |  |  |  | Time | 0.02 | 0.90 | 0.05 | 0.00 |
|  |  |  |  |  |  |  | Group*Time | 0.53 | 0.49 | 0.10 | 0.06 |
| Note.  Values are mean ± SD. P(obs) = Observed power, ^A^ = Computed using .05 alpha, **Δ** = Change, BMI = Body mass index, A/P = Anterior-posterior, M/L = Medial-lateral, 6MWT = Six-minute walk test, TUG = Timed up and go, STS = Sit-to-stand, 1RM = One-repetition Maximum. * = statical significance. | | | | | | | | | | | |
