# Supplementary material for: Comparison of low-dose maximal-intent versus controlled-tempo resistance training on quality-of-life, functional capacity, and strength in untrained healthy adults: a comparative effectiveness study
Source: BMC Sports Sci Med Rehabil. 2024 Mar 23;16:72. doi: 10.1186/s13102-024-00847-z (PMC10961002; doi:10.1186/s13102-024-00847-z)
Supplement: Supplementary file 3 — Supplementary Material 3. [file 13102_2024_847_MOESM3_ESM.docx]

| **Appendix 3.**  Shapiro-Wilk Test of Normality | | | | |
| --- | --- | --- | --- | --- |
|  |  | **Statistic** | **df** | **Sig.** |
| **Demographic** |  |  |  |  |
| **Mass, kg** | MI-Pre: | 0.93 | 10.00 | 0.44 |
|  | MI-Post | 0.90 | 10.00 | 0.22 |
|  | CT-Pre: | 0.90 | 10.00 | 0.22 |
|  | CT-Post | 0.89 | 10.00 | 0.19 |
| **BMI** | MI-Pre: | 0.82 | 10.00 | 0.03* |
|  | MI-Post | 0.83 | 10.00 | 0.04* |
|  | CT-Pre: | 0.85 | 10.00 | 0.07 |
|  | CT-Post | 0.88 | 10.00 | 0.13 |
| **Strength-to-mass** | MI-Pre: | 0.92 | 10.00 | 0.37 |
|  | MI-Post | 0.95 | 10.00 | 0.71 |
|  | CT-Pre: | 0.90 | 10.00 | 0.19 |
|  | CT-Post | 0.82 | 10.00 | 0.02 |
| **Leg Press 1RM, kg** | MI-Pre: | 0.94 | 10.00 | 0.56 |
|  | MI-Post | 0.87 | 10.00 | 0.09 |
|  | CT-Pre: | 0.96 | 10.00 | 0.79 |
|  | CT-Post | 0.91 | 10.00 | 0.31 |
| **Functional Capacity** |  |  |  |  |
| **TUGc, s** | MI-Pre: | 0.94 | 10.00 | 0.52 |
|  | MI-Post | 0.93 | 10.00 | 0.49 |
|  | CT-Pre: | 0.84 | 10.00 | 0.04* |
|  | CT-Post | 0.88 | 10.00 | 0.12 |
| **TUGa, s** | MI-Pre: | 0.94 | 10.00 | 0.60 |
|  | MI-Post | 0.91 | 10.00 | 0.30 |
|  | CT-Pre: | 0.82 | 10.00 | 0.03* |
|  | CT-Post | 0.92 | 10.00 | 0.36 |
| **6MWT, m** | MI-Pre: | 0.91 | 10.00 | 0.27 |
|  | MI-Post | 0.93 | 10.00 | 0.45 |
|  | CT-Pre: | 0.88 | 10.00 | 0.14 |
|  | CT-Post | 0.82 | 10.00 | 0.03* |
| **30sec STS, reps** | MI-Pre: | 0.92 | 10.00 | 0.35 |
|  | MI-Post | 0.87 | 10.00 | 0.10 |
|  | CT-Pre: | 0.85 | 10.00 | 0.07 |
|  | CT-Post | 0.96 | 10.00 | 0.78 |
| **Balance** |  |  |  |  |
| **BalanceO** | MI-Pre: | 0.78 | 10.00 | 0.01* |
|  | MI-Post | 0.99 | 10.00 | 0.99 |
|  | CT-Pre: | 0.92 | 10.00 | 0.35 |
|  | CT-Post | 0.97 | 10.00 | 0.92 |
| **BalanceAP** | MI-Pre: | 0.67 | 10.00 | 0.00* |
|  | MI-Post | 0.89 | 10.00 | 0.17 |
|  | CT-Pre: | 0.95 | 10.00 | 0.70 |
|  | CT-Post | 0.92 | 10.00 | 0.35 |
| **BalanceML** | MI-Pre: | 0.88 | 10.00 | 0.15 |
|  | MI-Post | 0.97 | 10.00 | 0.85 |
|  | CT-Pre: | 0.97 | 10.00 | 0.87 |
|  | CT-Post | 0.93 | 10.00 | 0.44 |
| Note.  BMI = Body mass index, A/P = Anterior-posterior, M/L = Medial-lateral, 6MWT = Six-minute walk test, TUG = Timed up and go, STS = Sit-to-stand, 1RM = One-repetition Maximum. Sig = significance. * = Statical significance. df = degrees of freedom. | | | | |
